# Residual Baculovirus in Insect Cell-Derived Influenza Virus-Like Particle Preparations Enhances Immunogenicity

**DOI:** 10.1371/journal.pone.0051559

**Published:** 2012-12-07

**Authors:** Irina Margine, Luis Martinez-Gil, Yi-ying Chou, Florian Krammer

**Affiliations:** 1 Department of Microbiology, Mount Sinai School of Medicine, New York, New York, United States of America; 2 Graduate School of Biological Sciences, Mount Sinai School of Medicine, New York, New York, United States of America; Pasteur Institute of Shanghai, Chinese Academy of Science, China

## Abstract

Influenza virus-like particles are currently evaluated in clinical trials as vaccine candidates for influenza viruses. Most commonly they are produced in baculovirus- or mammalian- expression systems. Here we used different vaccination schemes in order to systematically compare virus-like particle preparations generated in the two systems. Our work shows significant differences in immunogenicity between the two, and indicates superior and broader immune responses induced by the baculovirus-derived constructs. We demonstrate that these differences critically influence protection and survival in a mouse model of influenza virus infection. Finally, we show that the enhanced immunogenicity of the baculovirus-derived virus-like particles is caused by contamination with residual baculovirus which activates the innate immune response at the site of inoculation.

## Introduction

Vaccination remains the most effective counter measure against infection with influenza viruses [1], [2]. The seasonal influenza vaccines are effective when well matched with the circulating strain [3]. However, the occurrence of the swine-origin H1N1 virus in 2009 highlighted the need for fast and easy production strategies in the event of a pandemic. Influenza virus-like particles (VLPs) are evaluated as candidate vaccines, since they can be produced in a timely manner in response to a newly occurring influenza virus strain and they can efficiently present the hemagglutinin (HA) protein. Antibodies directed against this immunodominant influenza virus protein block entry and thereby inhibit viral replication.

Influenza VLPs have been produced in various expression systems including plants, mammalian cells, and most prominently, the baculovirus expression system [4]–[8]. VLPs expressed in the latter system are currently being tested in clinical trials in Mexico, and were recently reported to have yielded encouraging results in a phase II trial in 4563 healthy adults [9], [10]. General advantages of the baculovirus expression over mammalian cell culture-systems include higher yields, lower media costs and higher cellular growth rates. However, it is also associated with several disadvantages, such as non-mammalian-like protein glycosylation and the presence of high titers of contaminating baculovirus particles in the expression supernatants, especially when Sf9 cells are used which support the growth of baculovirus very well [11]. These contaminations are also found in VLP preparations used for vaccination since influenza VLPs and baculovirus virions have very similar densities and cannot be separated efficiently by density gradient ultracentrifugation [5], [11]–[14]. Mammalian cell-derived influenza VLPs are also being developed as vaccine candidates, and have proved successful in pre-clinical evaluations [6], [7], [15]. In the present study we compare influenza A VLPs produced in a mammalian and a baculovirus expression system, in terms of immunogenicity and protective ability in a mouse influenza virus infection model. Since generation of influenza VLPs has been described by co-expression of HA with the influenza matrix 1 (M1) protein [16], as well as with the retroviral Gag protein [12], we also evaluate the effect of the budding partner on immunogenicity. We find dramatic differences between the immune responses triggered by the different preparations in terms of anti-HA antibody titers, hemagglutination inhibition (HI) titers, antibody isotype profiles, cytokine induction by the antigen formulation and, finally, survival upon challenge with an influenza A virus. Interestingly, the differences in all these aspects correlate with the presence or absence of residual baculovirus in the preparations.

## Materials and Methods

### Cells, Plasmids and Viruses

Sf9 insect cells (ATCC# CRL-1711) were grown in TNM-FH media (Gemini Bioproducts) supplemented with 0.1% (w/v) Pluronic F-68 (Sigma), 100 U/mL penicillin and streptomycin (Gibco) and 5% (v/v) fetal calf serum (FCS). BTI-TN-5B1-4 (High Five) cells [11] were grown in Hyclone SFX media (Fisher Scientific). 293T and MDCK cells were obtained from the American Type Culture Collection (ATCC, Manassas, VA) and were maintained either in Dulbecco’s Modified Eagle Medium (DMEM) or in Minimum Essential Medium (MEM) (Gibco, Invitrogen) supplemented with 10% FCS (HyClone; Thermo Scientific) and penicillin and streptomycin (Gibco, Invitrogen). Mouse adapted influenza virus strain A/Puerto Rico/8/1934 (PR8) was grown in 10 day old embryonated chicken eggs for 2 days at 37**°**C and titered by plaque assay on MDCK cells. Expression plasmids encoding for M1, eGFP-Gag (Gag), PR8 HA and influenza B/Yamagata/16/1988 neuraminidase were constructed as described elsewhere [17]–[19].

### Recombinant Baculovirus Generation

Coding sequences for HA and M1 of PR8 and of eGFP-Gag were amplified by polymerase chain reaction (PCR) and cloned into a modified version of the baculovirus shuttle vector pFastBacDual (Invitrogen) using *Nhe*I and *Kpn*I (M1, eGFP-Gag) or *BamH*I and *Not*I (HA). Primer sequences are available upon request. Sequences were confirmed using Sanger sequencing. Recombinant vectors and empty vector (as control - referred to as ‘wild type’ herewith) were transformed into DH10Bac bacteria (Invitrogen) according to the manufacturer's recommendations; colonies with the desired phenotype were picked, propagated and bacmids were prepped using a Hipure Plasmid Filter Midiprep kit (Invitrogen). Bacmids were then transfected into Sf9 cells using Cellfectin II (Invitrogen) according to the manufacturer's recommendations. The rescued recombinant baculovirus was propagated and titered on Sf9 cells.

### VLP Generation, Purification and Quantification

Insect cell-derived VLPs were expressed as previously described [20]. Briefly, BTI-TN5B1-4 cells were co-infected at a multiplicity of infection of 10, either with baculoviruses that express HA and M1 or HA and eGFP-Gag, or with eGFP-Gag only (control VLPs). Infected cells were cultured in shaker flasks at 28**°**C at 80 rpm in a rotation shaker. Cultures were harvested 72 hours post-infection and supernatants were cleared by low-speed centrifugation at 2000 g at room temperature for 20 min. For the production of mammalian cell-derived VLPs, transient polyethylenimine (PEI) mediated transfections of the expression plasmids expressing either HA, M1 and influenza B neuraminidase, HA, eGFP-Gag and influenza B neuraminidase, or eGFP-Gag alone, were performed on 60–70% confluent 293T human embryo kidney cell layers. 1.1×10^7^ 293T cells were seeded in a 175 cm^2^ Roux flask and 24 hours later were transfected with PEI (Sigma-Aldrich) combined with the desired plasmid DNAs (45 µg total DNA per flask), at a 10∶1 ratio, as previously described [21]. Cleared supernatants from insect or 293T cultures were laid over 30% sucrose cushions and spun at 136,000 g, for 90 minutes at 4**°**C, and the pellet containing the VLPs was resuspended in phosphate buffered saline (PBS, pH 7.4). To determine the amount of HA protein in the different VLP preparations, we performed a Western blot followed by densitometric analysis (Image J, National Institutes of Health), using a purified recombinant PR8 HA [22] of known concentration as a standard. Western blot analysis was performed as previously described [23], by using the monoclonal antibody PY102 [24] to detect the PR8 HA (1∶1000 dilution) and a horseradish peroxidase-conjugated anti-mouse IgG secondary antibody (1∶5000 dilution). VLPs were further characterized as described before [11], [20] (Supplemental Material S1).

### Vaccination

This study was carried out in full compliance with the guidelines of the Mount Sinai School of Medicine Institutional Animal Care and Use Committee (IACUC). The protocol was approved by the Mount Sinai School of Medicine IACUC (animal protocol # LA12-00028). Procedures (vaccination, virus inoculation, etc.) were performed under ketamine/xylazine anesthesia (0.15 mg ketamine and 0.03 mg xylazine per mouse), and all efforts were made to minimize suffering. For the immunogenicity studies, groups of female, six- to eight-week old BALB/c mice were primed either intraperitoneally (i.p.) or intramuscularly (i.m.) with unadjuvanted mammalian cell- or baculovirus-derived eGFP-Gag or M1 driven VLPs harboring 500 ng of HA. Three weeks post prime, the animals primed via i.p. immunization were boosted i.p. with 500 ng of HA in the form of homologous VLPs. Animals that received an i.m. prime were either boosted i.m. or intranasally (i.n.) with equal amounts (500 ng of HA) of homologous VLPs under anesthesia. Group size was n = 3 for all immunogenicity studies except for the i.m./i.p. administered mammalian HA and M1 VLPs were the group size was n = 1. The control mice received similar amounts of either mammalian- or baculovirus-derived VLPs containing only eGFP-Gag, in an analogous manner (i.p. - i.p.). On day 42, mice were anesthetized and blood was collected by terminal cardiac puncture, followed by immediate sacrificing. Serum was separated from blood cells by performing a brief centrifugation and was stored at −20°C until further use.

In order to see differences in protection studies between the two immunogens we used a constant challenge dose (100 LD50) and titered down the vaccine dose. At a vaccine dose of 50 ng HA some morbidity in animals vaccinated with the baculovirus produced VLPs was observed and this amount of immunogen was then used for the comparative challenge as follows: Six- to eight-week old female BALB/c mice (n = 5) were vaccinated (i.m.) with mammalian- or baculovirus- derived, M1 driven VLPs containing 50 ng of HA, or were mock vaccinated with PBS. Three weeks post-vaccination, the mice were intranasally infected, while under anesthesia, with 100 mouse lethal dose 50 (mLD50) of PR8 virus. Weight was monitored daily for a period of 14 days, and mice losing more than 25% of their initial body weight were sacrificed and scored as dead.

### Enzyme Linked Immunosorbent Assay

96-well ELISA plates (Immulon 4 HBX) were coated with 0.2 µg per well of PR8 virus or with 0.1 µg per well of purified recombinant HA protein in carbonate coating buffer (100 mM, pH 9.6). PR8 virus used for this purpose was prepared from allantoic ﬂuid by concentration through a 30% sucrose cushion. The recombinant HA proteins of the A/Puerto Rico/8/1934, A/California/04/2009, A/Viet Nam/1203/2004 isolates, as well as the cH6/1 protein, were purified in the baculovirus system, as previously described [25]. Plates were blocked for one hour at room temperature with 3% milk in PBS. Afterwards, threefold serial dilutions of mouse sera were incubated on the coated plates, for two hours at room temperature. After extensive washing with PBS containing 0.1% Tween-20 (v/v), the bound antibody was detected with an alkaline phosphatase-linked anti-mouse IgG antibody (Invitrogen) (1∶5000) for one hour at RT, followed by another extensive washing step and detection with p-nitrophenyl phosphate (PNPP) substrate (Sigma). Optical density measurements were taken at 405 nm. For detection of IgA antibodies in nasal washes, a similar assay was employed, except that we used an alkaline phosphatase-linked anti-mouse IgA antibody (Southern Biotech) diluted 1∶500 and all incubations were performed at 37**°**C for three hours. Analysis of the isotype distribution in sera was determined by performing an ELISA assay, using recombinant PR8 HA as a substrate and an isotyping kit (Invitrogen), which contains a collection of secondary antibodies specific for each subtype and an AP-conjugated tertiary antibody that allows for detection of binding.

### Hemagglutination Inhibition Assay

PR8 virus was diluted to eight chicken erythrocyte hemagglutination units (3 wells), and 25 µl of this virus dilution was pre-incubated with 25 µL of two-fold dilutions of inactivated antisera [26]. After incubating for one hour on ice, 50 µl of a 0.5% chicken erythrocyte suspension was added to the mixture which was then incubated on ice for an additional hour. Virus mixed with PBS was used as a negative control, while PBS with no virus and no sera served as a background control.

### Detection of Interferon β and Mx1 Following Vaccination

For measurement of cytokine induction following vaccination, groups of six- to eight-week old BALB/c mice (n = 3) were immunized either i.n. or i.m. (calf muscle) with mammalian- or baculovirus-derived, M1 driven VLPs containing 500 ng of HA. Additionally, control mice received either PBS or a dose of purified wild-type baculovirus containing similar titers of infectious particles as the ones present in the administered dose of baculovirus-derived VLP preparation (4.5×10^6^ PFU/dose). Six hours post-inoculation, the calf muscle or the lungs were harvested and homogenized in 1 mL of Trizol-LS (Invitrogen) using a FastPrep-24 instrument (MP). The homogenized samples were passed through a QIAshredder column (Qiagen) and the RNA was extracted according to the manufacturer's protocol. Residual DNA in the samples was removed using the DNA-free kit (Ambion). To ensure for optimal RNA quality, the samples were subjected to treatment with the RNeasy kit (Qiagen). Complementary DNA was synthesized using oligoT primers and SuperScript III Reverse Transcriptase (Invitrogen) according to the manufacturer's protocol. Real-time PCR was performed using SYBR Green mix (Roche) and a LightCycler 480 Instrument (Roche). The sequences of the primers used for amplification are available upon request.

## Results

### Baculovirus-derived VLPs Induce a Stronger and Broader Immune Response Compared to Mammalian Cell-derived VLPs

To compare mammalian- and baculovirus-derived VLPs in terms of immunogenicity, we vaccinated BALB/c mice using a prime and boost regimen with VLPs containing 500 ng of PR8 HA. We compared VLPs that were made by co-expression of HA with either eGFP-Gag or M1, and administered them i.p./i.p., i.m./i.m. or i.m./i.n. We first wanted to confirm if, and to what extent, animals from the differently vaccinated groups seroconverted. Because of possible non-specific binding of sera from baculovirus-vaccinated animals to baculovirus-expressed proteins used for ELISA assays, we compared levels of reactivity in sera to both recombinant PR8 HA and purified PR8 virus.

**Figure 1:**
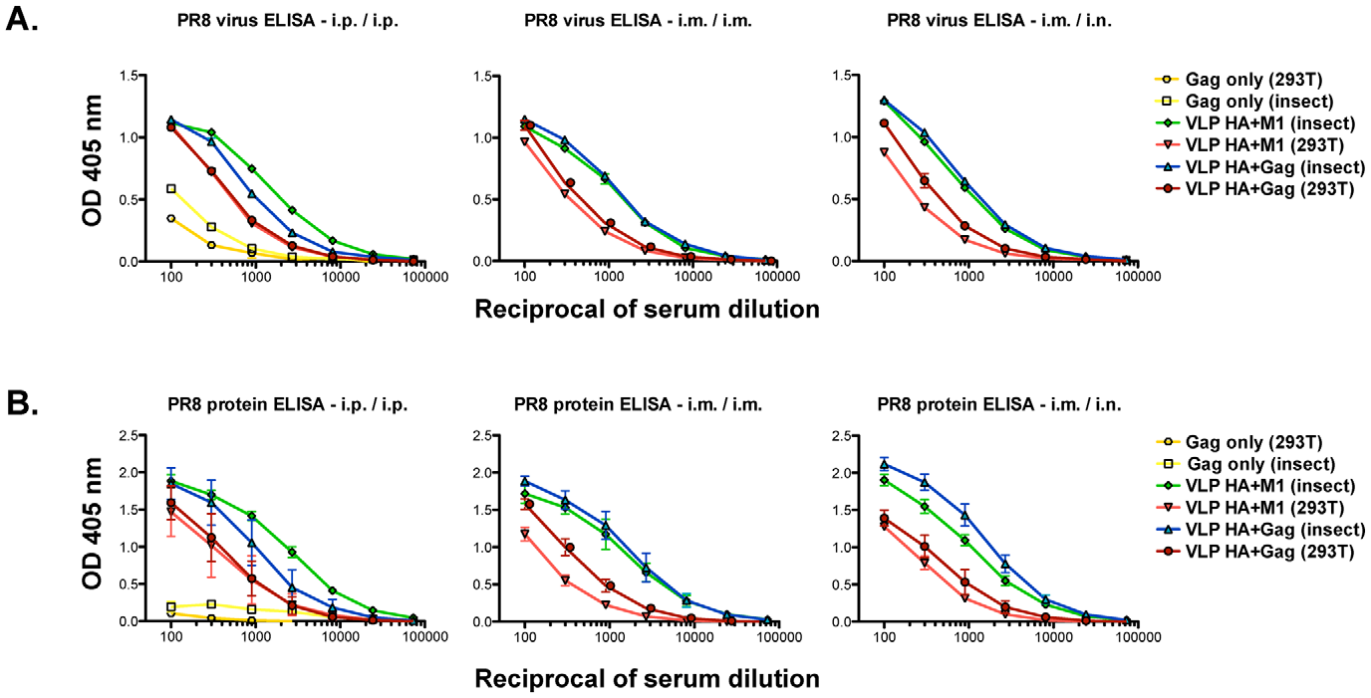
Seroconversion in mice immunized with different vaccine preparations. Pooled sera (**A**) or sera from individual mice (**B**) vaccinated by different immunization schemes with either mammalian cell- or baculovirus-derived VLPs, were tested for binding to purified PR8 virus or recombinant PR8 HA, respectively.

Irrespective of the vaccine protocol, we saw a significant difference in reactivity between sera of mice vaccinated with mammalian cell- or baculovirus-derived VLPs although all animals sero-converted to HA. Sera from animals vaccinated with preparations obtained in the latter system, whether M1 and eGFP-Gag driven, had significantly higher reactivity against both substrates. The highest titers were raised by the i.p./i.p. immunization scheme compared to the i.m./i.m. and i.m./i.n. regimens. No significant difference was appreciated in terms of immunogenicity induced by M1 versus eGFP-Gag driven VLP preparations. It is of note that sera from mice vaccinated with eGFP-Gag only VLPs (mammalian- and baculovirus-derived) had very low unspecific reactivity against the purified PR8 HA preparation in comparison to whole virus substrate (Figure 1A and B). Therefore, we decided to use purified HAs for all subsequent analysis but included sera from control animals vaccinated with HA-negative eGFP-Gag VLPs in all assays we performed.

To measure levels of HA head domain-directed antibody titers, we performed an HI assay with PR8 virus and sera obtained from the vaccinated animals and found similar trends as those seen in ELISA. The i.p./i.p. administration was able to induce the highest titers of hemagglutination inhibiting antibodies, followed by the other two vaccination regimens. A higher geometrical mean titer was observed for baculovirus-derived VLPs in general, although this finding is not significant for all cases. We also observed a higher variation in terms of HI activity in sera from mice vaccinated with mammalian cell-derived VLPs, compared to their baculovirus VLP vaccinated counterparts. No significant differences were observed between eGFP-Gag and M1 driven VLPs. All sera from control mice that received eGFP-Gag only VLPs tested negative (Table 1).

**Table 1:** Geometric means of hemagglutination-inhibition titers against homologous virus (PR8) induced by vaccination with mammalian- or baculovirus- derived VLP preparations.

HA+M1 Insect	HA+M1 293T	HA+Gag insect	HA+Gag 293T
100.79 (80–160^1^)	14.73 (1–80)	160 (160–160)	56.58 (40–80)

1 numbers in brackets represent the range of hemagglutination-inhibition values detected in sera of individual animals in each group.

**Figure 2:**
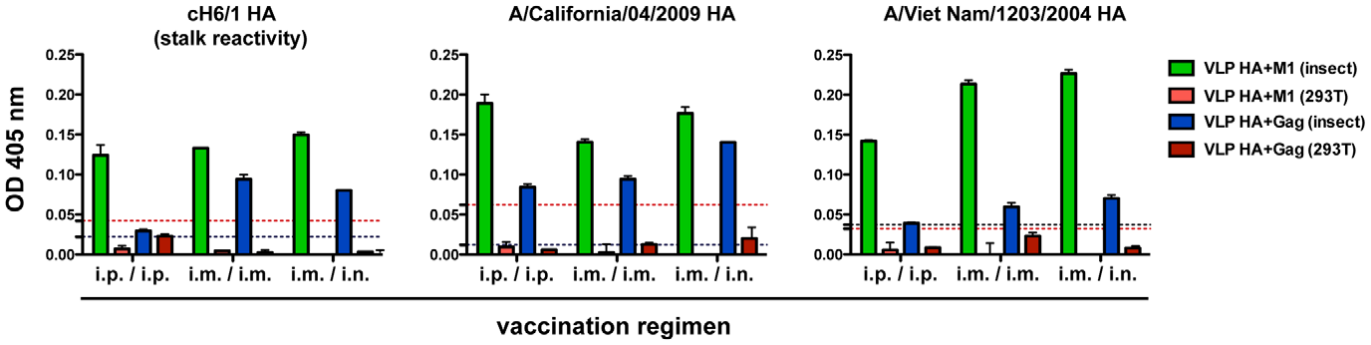
Assessment of cross-reactive activity in sera of mice immunized with different VLP preparations. Pooled sera from animals vaccinated with either mammalian cell- or baculovirus-derived VLPs were tested in ELISA for the ability to bind recombinant chimeric H6/1 (cH6/1 HA), H1 (A/California/04/2009 HA) or H5 (A/Viet Nam/1203/2004 HA) hemagglutinin. Data shown are from serum samples diluted 1∶300. Baselines represent background levels detected in this assay, when testing sera from animals vaccinated with HA negative, eGFP-Gag containing, mammalian cell- (red) or baculovirus-derived (black) VLPs.

Because of the increasing interest in the field to develop vaccine immunogens that provide broad protection to a variety of influenza virus subtypes, we next wanted to test reactivity to other group 1 HAs, as well as to the PR8 stalk domain. Antibodies with specificities against the highly conserved stalk domain of the HA glycoprotein have been described to have broadly reactive and cross-neutralizing characteristics [27]–[32]. For these purposes, we tested serum reactivity against HA proteins coming from the Cal09 and VN04 viral isolates, and a chimeric H6/1 HA [25], respectively. The highest antibody titers against the Cal09 substrate were found in sera from groups that received baculovirus-derived VLPs containing M1 and HA, followed by eGFP-Gag plus HA VLPs (Figure 2). Interestingly, vaccination with mammalian cell produced VLPs did not induce reactivity above background. Similar results were obtained when testing binding activity against VN04 or cH6/1 HA proteins. Our findings suggest that HA-M1 VLPs are better at inducing antibodies against the stalk domain of the influenza virus HA.

### Baculovirus-derived VLPs Trigger Preferable Antibody Isotype Profiles

**Figure 3:**
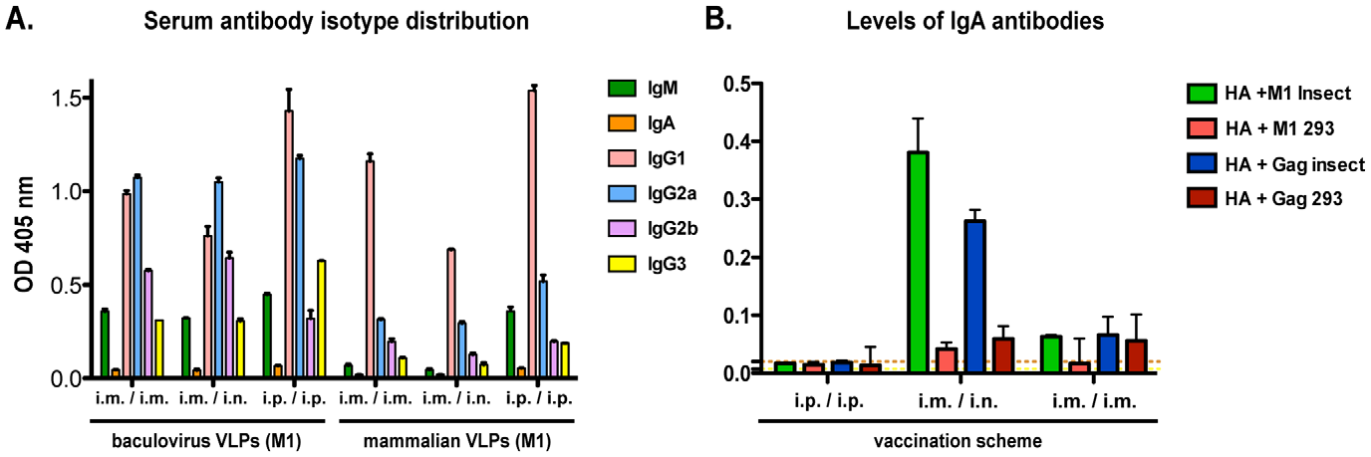
Antibody isotype profile in serum and mucosal samples from vaccinated animals. (**A**) Serum antibody isotype profile induced by different VLPs and immunization regimens. Serum was collected 3 weeks after the second immunization and analyzed for relative distribution of immunoglobulin subclasses by ELISA assay, using a recombinant PR8 HA protein as substrate and different secondary antibodies recognizing the different Ig isotypes. Signal was detected using a tertiary antibody coupled to an alkaline phosphatase (AP) enzyme. (**B**) Mucosal IgA antibody levels induced by the different vaccination regimens as analyzed by testing binding activity in nasal washes obtained from animals three weeks after the second immunization. Samples were pooled and binding was detected using an AP-coupled secondary antibody specific for the IgA subtype. Baselines represent background levels detected in this assay, when testing sera from animals vaccinated with HA negative, eGFP-Gag containing, mammalian cell- (orange) or baculovirus-derived (yellow) VLPs.

Next we analyzed the serum antibody isotype distribution, as a measure of the level of affinity maturation triggered by the different vaccination regimens, and also as a potential estimation of the neutralizing efficiency of the induced antibody responses. For this purpose, we compared relative levels of IgM, IgA and IgG subtypes in sera of mice immunized by different protocols with M1 containing baculovirus- or mammalian cell-derived VLPs using an ELISA. Expectedly, we detected an overall increased level of all serotypes in the mice vaccinated with insect cell-derived preparations (Figure 3A). Interestingly, we detected similar levels of IgM antibodies in the differently vaccinated groups that received baculovirus-derived VLPs, indicating similar levels of initial seroconversion, while the mammalian cell produced VLPs only induced significant levels of this class of antibodies when administered i.p./i.p. However, all groups underwent somatic maturation of serum antibodies, since differentiated IgG-types were present in all groups of mice, albeit at much lower levels in sera of mice who had received mammalian cell-derived VLP vaccines by i.m./i.m. or i.m./i.n. administration. When looking at the IgG subtype distribution, we saw similar levels of IgG1 type antibodies irrespective of the expression system in which the VLPs were produced. However, the baculovirus-derived VLPs produced significantly higher levels of IgG2a and IgG2b antibodies in all of the vaccination protocols assessed. These subclasses of IgGs are known to be the most pro-inflammatory IgG subtypes in mice and have been described to have a higher biological activity than IgG1 or IgG3 subtypes [33].

Next, we assessed the effect of the vaccination route on the generation of mucosal IgA. Baculovirus-derived VLPs have been previously reported to induce an IgA antibody type response in lung extracts of vaccinated mice, when administered i.n. [34]. We were particularly interested in the levels of IgA antibodies in the nasal washes of the animals, since these are known to play an important role in minimizing infections with influenza virus by neutralizing infectious particles in the respiratory tract [35]. Interestingly, we were indeed able to detect HA reactive IgA in samples from animals that received baculovirus-produced VLPs (both M1- and eGFP-Gag-derived) i.m./i.n., but not in sera from animals similarly immunized with mammalian cell-derived VLPs (Figure 3B).

### Residual Baculovirus in VLP Preparations Stimulates Innate Immune Responses

**Figure 4:**
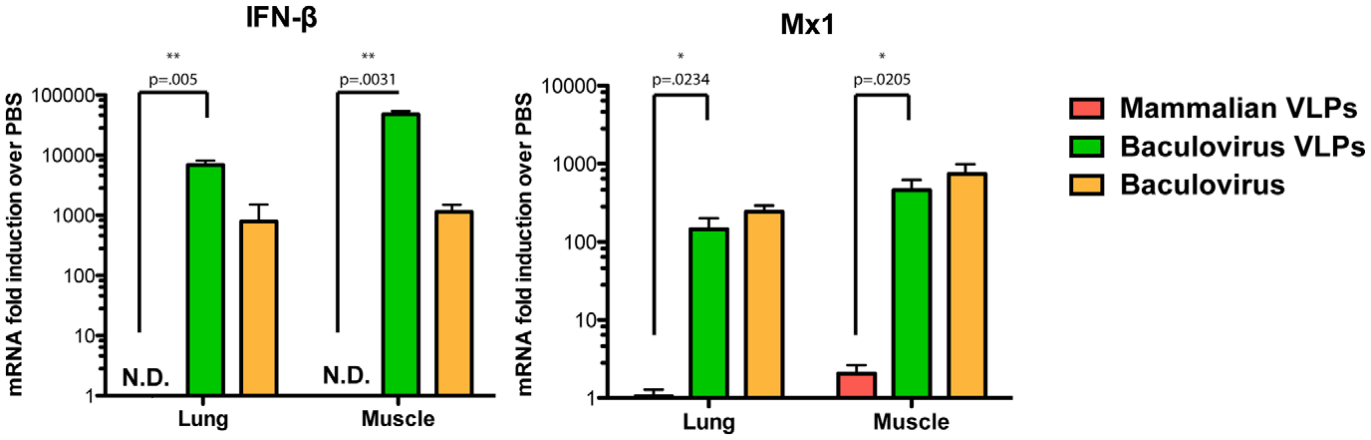
Assessment of innate response activation by different VLPs or purified baculovirus. Mice were immunized (intramuscularly or intranasally) with one dose of vaccine or the corresponding dose of wild type baculovirus (in PFU/dose) and 6 hours post-vaccination lung or muscle tissue was harvested and the level of induction of gene specific mRNA for IFN β, and Mx1 was analyzed as fold induction over tissue samples from animals mock-immunized with PBS.

Considering the significant differences in quantity and quality of the immune responses induced by vaccinations with baculovirus- and mammalian cell-derived VLP preparations, we further investigated the possible causative factors. Baculoviruses do not replicate in mammalian cells [36], but they have been reported to enter these cells and trigger an innate immune response, most probably through activation of TLR9 [5], [37]–[40]. We hypothesized that residual baculovirus in the insect cell-derived VLPs may activate the innate immune response, which may in turn be responsible for the observed enhanced immunogenicity of these preparations. When examining the baculovirus content in terms of plaque forming units (PFU) found in our VLP preparations, we detected 4.5×10^6^ PFU of baculovirus per vaccine dose, which is in the range of previous reports [5], [12], [13]. To investigate the effect this residual baculovirus may have on cytokine and interferon stimulated gene (ISG) induction, we compared mammalian cell-derived VLPs, baculovirus-derived VLPs and purified wild-type baculovirus. We inoculated mice either i.n. or i.m. with preparations of mammalian cell-derived VLPs, baculovirus-derived VLPs (both M1 triggered), a corresponding dose of purified baculovirus (corresponding PFU as in the baculovirus-derived VLP dose) and a PBS control. Six hours post-inoculation we harvested lungs or muscle and performed quantitative PCR analysis to measure mRNA levels of interferonβ and Mx1 genes. Interestingly, the baculo-derived VLPs and baculovirus, but not the mammalian VLPs were able to induce IFN**β** and Mx1 (Figure 4). This finding strongly suggests that the baculovirus contaminations in the insect cell preparations can induce innate immunity, and thereby have a potential immuno-enhancing effect for the antigen preparation [38], [41]. The lower levels of IFNβ induction determined for the purified baculovirus, when compared to the baculovirus-derived VLPs, may be explained by the wild type purified baculovirus having a lower PFU-to-defective interference (DI) particle ratio than baculovirus expressing recombinant proteins. Therefore, the number of baculovirus particles might be higher in the VLP vaccine than in the purified preparation, despite similar PFUs [42].

### Enhanced Protection by Baculovirus-derived VLPs in Comparison to Mammalian-derived VLPs

**Figure 5:**
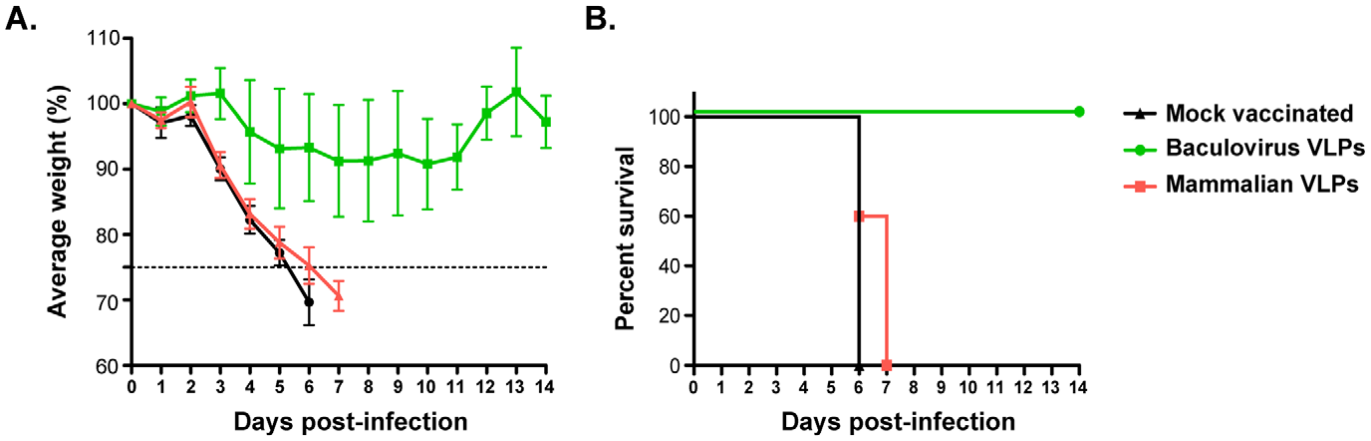
*In vivo* protection by vaccination with VLP preparations generated in either mammalian or insect cells. Mice were vaccinated once with mammalian cell-, or baculovirus- derived VLPs containing 50 ng of HA, and were intranasally infected with 100 mLD50 of PR8 virus three weeks post vaccination. (**A**) Body weight loss was monitored daily for 14 days and (**B**) animals that lost more than 25% of their initial body weight were scored dead (Kaplan-Meier curve).

Finally, we assessed the protective effect of the enhanced immunogenicity shown by the baculovirus-derived VLPs in the context of an *in vivo* infection. In order to detect differences in protection, we chose a low single immunization dose (50 ng of HA in HA plus M1 VLPs) that was found to be the lowest protective dose (from mortality) for baculovirus-derived VLPs in a challenge experiment with 100 mLD50 of PR8 virus (data not shown). Mice were immunized once, intramuscularly, with 50 ng of HA in form of M1 driven, mammalian- or baculovirus-derived VLPs. We chose this vaccination route since i.n., but not i.m. immunization with wild-type baculovirus protects from viral challenge through stimulation of innate immunity [43]. Three weeks post immunization, mice were challenged intranasally with 100 mLD50 of PR8 virus. The mice that received this low dose of mammalian cell produced VLPs lost weight with similar kinetics as the mock-vaccinated group, and succumbed to disease caused by the virus infection by day 7 (Figure 5). Animals that received baculovirus produced VLPs however, only lost approximately 15% of their body weight, looked otherwise healthy (scores not shown), regained their initial body weight quickly and survived the viral infection. We therefore conclude that the differences in serum antibody levels translate to enhanced *in vivo* protection in a mouse influenza virus infection model.

## Discussion

In this study we compared influenza A VLPs produced in a mammalian and a baculovirus expression system, in terms of immunity and protection induced in a mouse model of influenza virus infection. We detected a significantly higher immunogenicity induced by the baculovirus-derived VLPs in terms of homologous- and heterologous- HA specific IgG and IgA antibody titer, and a similar trend in HI titers although mice sero-converted to both immunogens. Furthermore, the baculovirus-derived VLP preparations significantly biased the isotype distribution of the antibody response towards the more active IgG2 immunoglobulin subtype. Additionally we observed that M1, but not eGFP-Gag driven particles are able to induce a small but significant antibody response directed to the stalk domain of the PR8 HA. These antibodies have recently been described to have broadly neutralizing abilities, and vaccination strategies that can efficiently induce them are considered to be promising for the development of a universal influenza virus vaccine [44].

In various assays, the M1-derived VLPs were found to be more immunogenic when compared to eGFP-Gag driven preparations. It is possible that by coupling HA with a natural budding partner, M1-derived VLPs may be more immunogenic due to preferential antigen uptake, receptor binding by the VLPs, fusion with the endosomal membrane, or antigen presentation. This was suggested by previous studies that detected broader reactivity in baculovirus-derived VLP vaccines containing M1 [45]–[47]. Furthermore, we showed that this enhanced immunogenicity translates well into protection from lethal influenza virus challenge in a single-dose immunization model. We hypothesize that residual baculovirus particles in the VLP preparations are responsible for their enhanced immunogenicity, and we show that baculovirus-derived VLPs, as well as wild type baculovirus, induce high levels of interferon at the site of inoculation. This induction may trigger the recruitment of innate immune cells, as well as activation and maturation of dendritic cells that produce inflammatory cytokines and present antigen to B cells. Collectively, these processes could be responsible for the observed effect. Along these lines, a recent report interestingly showed that MyD88^−/−^ mice have a diminished response to vaccination with baculovirus-derived VLPs, as compared to wild-type mice [48]. Actually, the pattern demonstrated in MyD88^−/−^ mice resembles the response we observed with mammalian cell-derived VLPs and supports our hypothesis, since baculovirus induced signaling occurs mainly through activation of TLR9, and MyD88 downstream [37], [39], [49].

Our results therefore have practical implications. Since baculovirus is hard to separate from influenza VLPs due to the similar density and enveloped nature they share, it is always present in “purified” VLP preparations [5], [11]–[14]. We hypothesize that the enhanced immunogenicity seen with baculovirus-expressed influenza VLPs in animal models is dependent on the presence of these viral contaminations. As a consequence, pre-clinical results with non-inactivated preparations may be hard to translate into clinical trials where these preparations have to be inactivated because of safety concerns [10]. Inactivation of baculoviruses with different chemical (binary ethylenimine, Triton X100) or physical (UV irradiation) reagents has been shown to abolish the interferon-inducing and immuno-enhancing ability of baculovirus impurities [38]. The presence of active baculovirus particles in vaccine preparations might lead to enhanced reactogenicity including local inflammation, soreness and fever. Additionally, there is a general concern by regulatory agencies about live, contaminating viruses or virus genomes in human vaccines.

In summary, we demonstrate the higher immunogenicity of baculovirus-derived VLPs compared to mammalian-cell based systems in terms of eliciting of differentiated IgG and also mucosal IgA responses. These differences can be directly translated into protection in an *in vivo* model of influenza virus infection. The enhanced immunogenicity of the insect cell-derived influenza VLPs can be explained by residual, contaminating baculovirus that induces a local antiviral response.

## Supporting Information

Supplemental Material S1 (PDF)
