# Supplementary material for: Residual Baculovirus in Insect Cell-Derived Influenza Virus-Like Particle Preparations Enhances Immunogenicity
Source: PLoS One. 2012 Dec 7;7(12):e51559. doi: 10.1371/journal.pone.0051559 (PMC3517492; doi:10.1371/journal.pone.0051559)
Supplement: Supplemental Material S1 — (PDF) [file pone.0051559.s001.pdf]

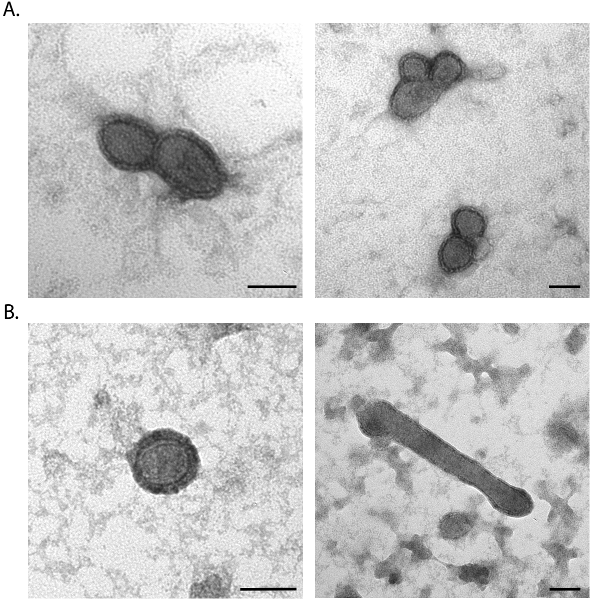

Figure S1. Transmission electron microscopy examination of different VLP preparations. VLP preparations were subject to negative staining using 1% aqueous uranyl acetate followed by TEM examinations with HITACHI 7700 at 80kV. (A) Transmission electron micrographs of VLPs (M1 driven) derived from mammalian 293T cells are shown. (B) Transmission electron micrographs of baculovirus-derived VLPs (M1 driven) are shown, the left image shows the morphology of the VLP and the right image shows the presence of baculovirus in the VLP preparations. Scale bar=50 nm.

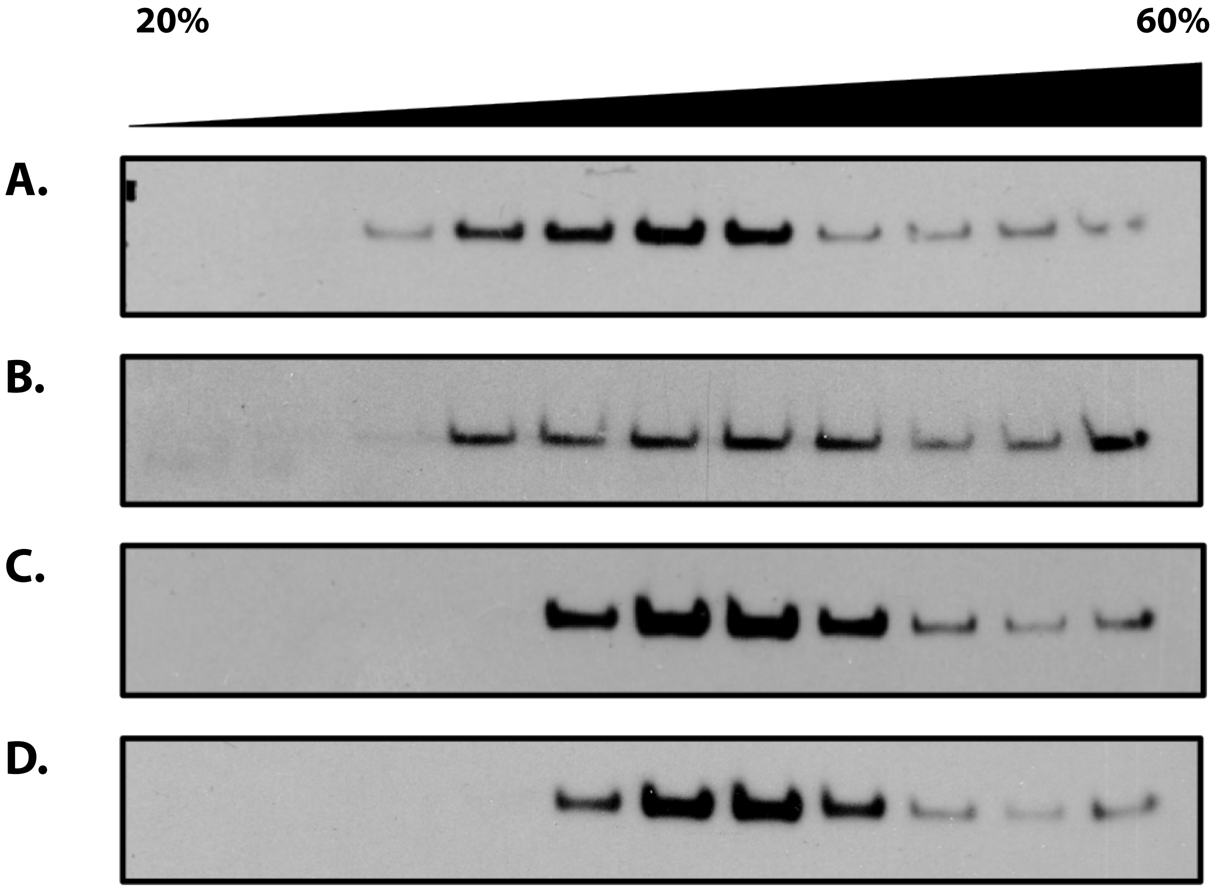

Figure S2. Sucrose density gradient analysis of VLP preparations. Cleared supernatants from insect or 293T cultures were laid over 30% sucrose cushions and spun at 136,000 g, for 90 minutes at 4°C, and the pellet containing the VLPs was re-suspended in phosphate buffered saline (PBS, pH 7.4). VLPs were then loaded onto a discontinuous sucrose density gradient (20%, 30%, 40%, 50%, 60%) and spun at 190 000 × g at 4°C for 16 h. Gradients were split into 11 fractions, which were analyzed by SDS-PAGE (on 4-16% polyacrylamide gels) and Western blot. HA was detected using mouse monoclonal antibody PY102 (produced in-house). Insect cell-derived VLPs made by co-expression of HA and eGFP-Gag are shown in (A), M1-driven ones are shown in (B). Mammalian cell derived VLPs made by co-expression of HA, influenza B neuraminidase and eGFP-Gag are shown in (C) and M1-driven mammalian cell-derived VLPs are shown in (D).
